# Corbella Extra Virgin Olive Oil, Urinary Phenolic Metabolites, and Exploratory Cardiometabolic Outcomes in Healthy Young Adults: The HEVOOC Trial

**DOI:** 10.3390/antiox15081033

**Published:** 2026-08-19

**Authors:** Rocío M. Gutiérrez-Romero, Carolina Donat-Vargas, Polina Galkina, Camila Arancibia-Riveros, Inés Domínguez-López, Sara Hurtado-Barroso, Maria Pérez, Anna Vallverdú-Queralt, Rosa Casas, Ramón Estruch, Rosa M. Lamuela-Raventós

**Affiliations:** 1 Polyphenol Research Group, Department of Nutrition, Food Sciences and Gastronomy, Faculty of Pharmacy and Food Sciences, Universitat de Barcelona (UB), 08036 Barcelona, Spain; rogutierrez@ub.edu (R.M.G.-R.); carolina.donat@ub.edu (C.D.-V.); polinagalkina@ub.edu (P.G.); camila.arancibia@gmail.com (C.A.-R.); mariaperez@ub.edu (M.P.); avallverdu@ub.edu (A.V.-Q.); 2 Institut de Recerca en Nutrició i Seguretat Alimentària (INSA-UB), Universitat de Barcelona (UB), 08921 Santa Coloma de Gramenet, Spain; ines.dominguez-lopez@dife.de (I.D.-L.); rcasas1@recerca.clinic.cat (R.C.); restruch@ub.edu (R.E.); 3 Epidemiology and Public Health Networking Biomedical Research Centre (CIBERESP), Instituto de Salud Carlos III, 28029 Madrid, Spain; 4 Unit of Cardiovascular and Nutritional Epidemiology, Institute of Environmental Medicine, Karolinska Institutet, 17177 Stockholm, Sweden; 5 CIBER Fisiopatología de la Obesidad y Nutrición (CIBEROBN), Instituto de Salud Carlos III, 28029 Madrid, Spain; shurtado@uic.es; 6 Department of Molecular Epidemiology, German Institute of Human Nutrition Potsdam-Rehbruecke, 14558 Nuthetal, Germany; 7 Department of Biomedical Science, Faculty of Medicine and Health Science, Universitat Internacional de Catalunya, 08195 Sant Cugat del Vallès, Spain; 8 Department of Internal Medicine, Institut d’Investigacions Biomèdiques August Pi Sunyer (IDIBAPS), Hospital Clínic, Universitat de Barcelona (UB), 08036 Barcelona, Spain

**Keywords:** *Olea europaea*, Mediterranean diet, (poly)phenols, hydroxytyrosol, HbA1c, cardiometabolic health, glycemic control, central adiposity, urinary phenolic metabolites, Corbella

## Abstract

Extra virgin olive oil (EVOO) is a hallmark of the Mediterranean diet and an important dietary source of bioactive phenolic compounds, including hydroxytyrosol and tyrosol derivatives, which have been investigated in relation to cardiometabolic health. However, the potential metabolic effects of phenolic-rich EVOO in young healthy adults remain poorly characterized. In this randomized crossover trial, 28 healthy adults (29.0 ± 3.4 years) consumed either phenolic-rich EVOO or regular olive oil (OO) (0.7 g/kg/day) for 4 weeks. The cardiometabolic outcomes reported here were secondary endpoints of the trial. Anthropometric measurements, cardiometabolic biomarkers, and urinary phenolic metabolites were assessed at baseline and after each intervention period; selected phenolic metabolites were quantified in 24-h urine using targeted LC–HRMS. Treatment effects were evaluated using mixed-effects models. Compared with OO, EVOO markedly increased urinary hydroxytyrosol-glucuronide (GMR = 9.9; 95% CI: 2.0, 49.0; *p* = 0.005), together with other hydroxytyrosol- and tyrosol-derived metabolites, providing objective evidence of greater phenolic exposure during the EVOO intervention. Exploratory between-intervention differences favored EVOO for waist circumference (β = −2.21 cm; 95% CI: −3.37, −1.05), waist-related indices, and HbA1c (β = −0.13%; 95% CI: −0.25, −0.01). In exploratory analyses, increases in urinary hydroxytyrosol-glucuronide during EVOO intake were inversely correlated with changes in HbA1c (Spearman’s rho = −0.49, *p* = 0.011) and WHR (rho = −0.38, *p* = 0.048). Post hoc sensitivity analyses showed greater stability for the waist-circumference estimate than for HbA1c. Overall, phenolic-rich ‘Corbella’ EVOO substantially increased urinary phenolic metabolite exposure, whereas the observed cardiometabolic differences should be considered exploratory and hypothesis-generating and require confirmation in larger trials specifically powered for these outcomes.

## 1. Introduction

The Mediterranean diet (MD) has been widely recognized for its health benefits, particularly in the prevention of cardiovascular and metabolic diseases [1,2]. Among its most characteristic components, extra virgin olive oil (EVOO) stands out as a central pillar of the MD and represents the principal culinary fat traditionally used for both cooking and dressing [3]. Population-based studies have consistently shown the cardiovascular and metabolic benefits associated with olive oil consumption [2].

Commercially available regular olive oil (OO) is a blend of refined and EVOO and contains relatively low concentrations of bioactive compounds. In contrast, EVOO retains significantly higher levels of bioactive phenolic compounds and other minor constituents than refined olive oil [4]. Despite these compositional differences, few epidemiological studies have been able to clearly distinguish the health effects of regular OO from those of EVOO [5].

Evidence from randomized clinical trials supports the metabolic benefits of EVOO consumption [6]. The PREDIMED trial demonstrated that adherence to an MD supplemented with EVOO reduced the incidence of type 2 diabetes by approximately 50% in individuals at high cardiovascular risk, an effect partially attributed to improvements in insulin sensitivity and glucose uptake mediated by oleic acid and EVOO phenolics [7]. Secondary analyses reported no increase in body weight and a tendency towards reduced central adiposity compared with a low-fat control diet [8]. Central adiposity is recognized as an early and sensitive marker of cardiometabolic risk, even in individuals with normal body weight and otherwise favorable metabolic profiles [9].

Beyond long-term prevention trials, shorter controlled interventions have provided mechanistic evidence supporting the effects of EVOO on glucose regulation. In individuals with impaired fasting glucose, EVOO improved postprandial glucose responses, incretin secretion, and insulin release [10], while in patients with type 1 diabetes, it attenuated early glucose excursions compared with butter or low-fat meals [11]. In overweight women, daily consumption of 25 mL of EVOO within a hypocaloric diet reduced body fat and diastolic blood pressure compared with soybean oil, highlighting potential benefits for body composition and vascular function [12].

The effects of phenolic-rich EVOO on lipid-related outcomes have been more extensively investigated. In the EUROLIVE multicenter trial, oils with higher phenolic content increased HDL cholesterol and reduced LDL oxidation in a dose-dependent manner [13]. Additional crossover interventions have reported improvements in endothelial function following consumption of high-polyphenolic EVOO [14,15]. In hypercholesterolemic participants, phenol-rich virgin olive oil improved ischemic reactive hyperemia and was accompanied by lower postprandial lipid peroxides and 8-epi-prostaglandin F2α and higher nitric oxide metabolite concentrations than low-phenol olive oil [15]. These findings suggest that the phenolic fraction of olive oil may support endothelial responsiveness by limiting oxidative stress and preserving nitric oxide bioavailability; however, these mechanisms were not assessed in the present trial. Nevertheless, a recent network meta-analysis including more than 7000 participants suggested that improvements in glucose, LDL cholesterol, and oxidized LDL are largely attributable to adherence to the MD as a whole, whereas the only direct effect directly linked to EVOO was an increase in HDL cholesterol [16]. Thus, the specific cardiometabolic contribution of EVOO, particularly in young and metabolically healthy populations, remains incompletely defined. Although some investigations have reported reductions in glucose levels following EVOO supplementation, these studies often lacked appropriate control groups, thereby restricting the strength of their conclusions.

Urinary hydroxytyrosol- and tyrosol-derived conjugates provide objective biomarkers of exposure to and metabolism of olive-oil phenolic compounds in human intervention studies [17]. Their urinary excretion reflects absorption, metabolism, and elimination of these compounds but does not, by itself, demonstrate antioxidant activity or reduced oxidative stress [17,18].

Most research to date has focused on widely cultivated commercial EVOO varieties, leaving recovered native cultivars relatively understudied. In the present study, we investigate ‘Corbella’ EVOO, a native Catalan variety characterized by a distinctive phenolic profile, particularly rich in oleuropein aglycon, which may contribute to its oxidative stability and potential health benefits [19].

Accordingly, this secondary analysis of the HEVOOC randomized crossover trial aimed to characterize the urinary exposure to selected olive oil phenolic metabolites and to explore short-term cardiometabolic responses to Corbella EVOO compared with low-polyphenol olive oil in young, metabolically healthy adults. This study seeks to advance understanding of the potential functional relevance of phenolic-rich EVOO in young and healthy individuals.

## 2. Materials and Methods

### 2.1. Study Design and Ethical Considerations

The HEVOOC (Healthy Extra Virgin Olive Oil Corbella) trial was a randomized, controlled, crossover clinical study conducted at Hospital Clinic of Barcelona (Spain) from June to September 2023. A pilot neuroimaging substudy involving nine participants from the HEVOOC cohort has previously been reported [20]. The present manuscript reports secondary cardiometabolic outcomes and urinary phenolic metabolites from the full cohort completing the trial (*n* = 28). The cardiometabolic variables presented in this manuscript correspond to prespecified secondary endpoints of the trial. The original sample size was calculated based on brain-derived neurotrophic factor (BDNF), with 80% power, a two-sided significance level of 5%, and an anticipated dropout rate of 20% [20]. No separate sample-size calculation was performed for the cardiometabolic outcomes.

The study protocol was reviewed and approved by the Ethics Committee of Hospital Clínic de Barcelona (HCB/2023/0074; 16 May 2023) and the Bioethics Commission of the University of Barcelona (IRB00003099; 4 July 2023). The trial was registered at ClinicalTrials.gov (National Library of Medicine, USA) with the identifier NCT05898113 on 12 June 2023 (URL: https://clinicaltrials.gov/study/NCT05898113, accessed on 12 August 2026). All participants provided written informed consent before participation. Patients and the public were not involved in the design, conduct, or reporting of this trial. All study procedures were conducted in accordance with the Declaration of Helsinki and Good Clinical Practice principles.

### 2.2. Participants

Male and female participants were recruited through online platforms, institutional networks, word of mouth, and posters displayed on campus and at the Hospital Clínic de Barcelona. Inclusion criteria were age between 18 and 35 years, BMI < 30 kg/m^2^, absence of chronic diseases, and non-smokers. Exclusion criteria included food allergies or intolerances, adherence to extreme or restrictive diets, excessive alcohol intake (>30 g/day for men and >20 g/day for women), pregnancy, menopause, or breastfeeding.

All volunteers who responded to the study advertisements were contacted and given information about the study design and procedures. Initial eligibility was assessed through a telephone screening and a brief digital questionnaire to verify basic inclusion criteria. The Alcohol Use Disorders Identification Test (AUDIT) [21] was used as a screening method to assess the level of risk of alcohol use. Potentially eligible participants were invited to an in-person screening visit, during which they completed a structured questionnaire covering medical history, physical activity, and dietary habits. Final eligibility was determined by the research team through application of the exclusion criteria during a face-to-face interview.

Participants who met all eligibility criteria were randomized using block-balanced randomization to ensure equal allocation across intervention sequences. Randomization was performed using an online tool (http://www.randomization.com/). Due to the nature of the intervention, both participants and investigators who administered the intervention were aware of group allocation, whereas external outcome assessors and data analysts were blinded to the assigned treatment.

### 2.3. Intervention and Trial Procedures

The study aimed to evaluate the effects of EVOO compared with regular OO on health-related parameters. Participants consumed either 0.7 g of EVOO or OO per kilogram of body weight daily for 4 weeks. Participants were instructed to maintain their habitual diet throughout the study, except for the dietary restrictions specified in the protocol. The assigned study oil was incorporated into participants’ usual meals and could be consumed raw or used for cooking, dressings, or spreads. Participants were advised to use the assigned oil as their only olive oil during each intervention period, although other dietary sources of fat were not prohibited. Both study oils were provided by the investigators in 5-L dark containers. Daily adherence records documented whether the assigned oil was consumed and whether it was used raw or cooked. Oil intake was not independently quantified by weighing returned containers or residual oil.

Following the first intervention period, participants underwent a 4-week washout phase before crossing over to the alternate intervention, allowing for direct within-subject comparison. The study design is illustrated in Figure S1.

Prior to the first intervention period, participants completed a 7-day run-in period during which they consumed only regular olive oil. Dietary restrictions aimed at minimizing background (poly)phenol exposure were maintained throughout the study. Olives were excluded throughout the entire study period. In addition, red fruits, apples, and oranges were avoided, and polyphenol-rich beverages were limited to minimize potential confounding from other dietary sources of polyphenols. Anthropometric measurements, biochemical markers, and urinary phenolic metabolites were assessed at baseline and at the end of each intervention phase.

### 2.4. Intervention Oils

Two olive oils were used in the clinical trial. Prior to the intervention, five commercially available regular olive oils were screened for total phenolic content using the Folin–Ciocalteu assay, expressed as gallic acid equivalents (GAE) per kg of oil. Three replicate determinations were performed for each oil. The oil with the lowest total phenolic content was selected as the control OO. Total phenolic content was 227.7 ± 19.7 mg GAE/kg for the ‘Corbella’ EVOO and 12.3 ± 0.5 mg GAE/kg for the selected regular OO.

The detailed phenolic profile of the ‘Corbella’ EVOO was determined by UPLC–MS/MS following liquid–liquid extraction based on Kalogeropoulos et al. [22]. Analyses were performed using an Acquity UPLC system (Waters, Milford, MA, USA) coupled to an API 4000 triple-quadrupole mass spectrometer (PE Sciex, Concord, ON, Canada), operated in negative electrospray ionization mode using multiple reaction monitoring (MRM). Major secoiridoids, including oleacein, oleocanthal, oleuropein aglycone, and ligstroside aglycone, were determined according to Lozano-Castellón et al. [23], whereas the remaining phenolic compounds were analyzed according to López-Yerena et al. [24]. Calibration curves were constructed using available authentic standards; compounds without commercially available standards were quantified using structurally related phenolic standards. Analyses were performed in triplicate. The detailed phenolic profile is provided in Table S1.

The fatty-acid profile of the ‘Corbella’ EVOO was determined by gas chromatography with flame-ionization detection (GC-FID) after fatty-acid methyl ester derivatization, following López-López et al. with minor modifications [25]. The fatty-acid composition of the control OO was obtained from a single determination performed by an external laboratory and is therefore reported without an SD. The fatty-acid composition of both intervention oils is presented in Table S2.

The ‘Corbella’ EVOO was specifically developed as the prototype oil for the subsequent clinical intervention. It was produced from ‘Corbella’ olives harvested on 6 and 13 October 2022, with the oils obtained from the two harvest dates combined to provide sufficient product for the subsequent clinical-study phase. As part of its development for the intervention, the EVOO prototype underwent longitudinal stability assessment at baseline and after 6 and 12 months of storage under bag-in-box and stainless-steel/N_2_ conditions. Oxidative stability was assessed using the Rancimat method [26], and free acidity, 1,2-diacylglycerols (DAGs), pyropheophytins (PPPs), and the phenolic profile were also evaluated. Shelf-life-related quality parameters were assessed according to Guillaume and Ravetti [27]. Stability analyses were performed in triplicate, and the corresponding data are presented in Table S3.

Participants received the assigned study oil at the beginning of each 4-week intervention period and recorded daily whether it was consumed raw or used for cooking.

### 2.5. Sample Collection

Clinical assessments were performed on four occasions during the study: before and after each intervention period. Follow-up visits took place at IDIBAPS—Hospital Clínic de Barcelona between 8 and 9:30 a.m., after an overnight fast. Fasting blood samples were collected at each visit.

Blood and 24-h urine were obtained at the beginning and the end of each intervention period. Plasma was separated by centrifugation at 2000 *g* for 10 min at 4 °C. Participants collected 24-h urine, and the total volume of urine collected was recorded. All biological samples were immediately aliquoted and stored at −80 °C until analysis.

### 2.6. Anthropometric, Blood Pressure, and Clinical Assessment

Assessments began with anthropometric measurements performed under fasting conditions, including height, weight, body fat mass, muscle mass, waist, and hip circumferences. Standing height was measured using a portable stadiometer. Body weight, fat mass, and muscle mass were estimated with an OMRON BF511 (OMRON Healthcare Co., Ltd., Kyoto, Japan) tetrapolar bioelectrical impedance analyzer, with participants wearing light clothing and no shoes.

Body mass index (BMI) was calculated as weight (kg) divided by height squared (m^2^). Waist circumference was measured at the midpoint between the lowest rib and the iliac crest by the same trained evaluator, and hip circumference at the level of maximal gluteal extension. The waist-to-hip ratio (WHR) was calculated as waist circumference divided by hip circumference (cm/cm). The waist-to-height ratio (WHtR) was calculated as waist circumference divided by height (cm/cm). Following anthropometric measurements, systolic and diastolic blood pressure and heart rate were measured in triplicate in the seated position using an OMRON M6 automated monitor. Measurements were taken after a brief rest period.

Finally, the clinical blood analyses were conducted at the CORE Laboratory of the Hospital Clínic.

### 2.7. Dietary Intake and Physical Activity

Diet and physical activity were assessed at baseline and at the end of each intervention period. Dietary intake was evaluated using a validated 151-item food frequency questionnaire [28]. The post-intervention FFQ assessed dietary intake during the preceding month corresponding to each intervention period and included consumption of the study oil supplied during that period. Three-day dietary records were additionally collected before the clinical assessments. Adherence to the MD was assessed using the 17-item Mediterranean Diet Adherence Screener (MEDAS) [29]. Each item was scored 0 or 1 according to compliance with the Mediterranean dietary pattern, yielding a total adherence score.

Objective physical activity was measured using ActiGraph^®^ wGT3X-BT (ActiGraph LLC, Pensacola, FL, USA) accelerometers worn on the non-dominant wrist for 7 consecutive days prior to each visit. Each device was programmed with the participant’s unique study ID. Data were analyzed using ActiLife software (v6.13.4). Moderate-to-vigorous physical activity (MVPA) was defined as ≥ 1952 counts per minute (cpm), according to the Freedson adult cut-points [30]. Daily MVPA was calculated as the average number of minutes per day spent above this threshold.

### 2.8. Urinary Metabolites

Urinary (poly)phenol metabolites were quantified in 24-h urine samples using liquid chromatography (Agilent 1290 Infinity II) coupled with high-resolution mass spectrometry (Agilent 6560 Ion Mobility QTOF LC/MS [Agilent Technologies, Santa Clara, CA, USA]), operated in negative electrospray ionization mode.

All analyses were conducted at the Separation Techniques Unit of the Scientific and Technological Centers (CCiTUB), Universitat de Barcelona. Sample preparation and analytical procedures were based on a previously described LC-HRMS protocol developed by our research group [31], with adaptations for the targeted hydroxytyrosol- and tyrosol-related metabolites analyzed in the present study. Quantification was performed in µg/L. For the metabolites included in the present study, the LOD/LOQ values were 0.373/1.242 µg/L for hydroxytyrosol-glucuronide, 0.244/0.813 µg/L for hydroxytyrosol-sulfate, 10.821/36.070 µg/L for tyrosol, and 0.417/1.392 µg/L for tyrosol-glucuronide. Values below the LOD for which a mass-spectrometric peak was detected were assigned LOD/2, whereas values between the LOD and LOQ were assigned the midpoint between the corresponding LOD and LOQ. When fewer than 5% of measurements for a compound exceeded the upper limit of quantification (ULOQ), these values were assigned the ULOQ; compounds with more than 5% of measurements above the quantification range were excluded from the analytical dataset.

Twenty-four-hour urine collection was used to obtain an integrated daily urine sample and to reduce variability related to the timing of a single spot-urine sample.

### 2.9. Statistical Analysis

Descriptive statistics are presented as means and standard deviations (SDs) for continuous variables, and as frequencies and percentages for categorical variables. The distribution of continuous variables was visually inspected using histograms and formally assessed for normality with the Shapiro–Wilk test. Variables with skewed distributions were log-transformed prior to analysis.

The main between-intervention inference for the cardiometabolic outcomes presented in this manuscript was based on linear mixed-effects ANCOVA models (LMM) including intervention, period, sequence, and the corresponding baseline value as fixed effects, with participant as a random intercept. Anthropometric and biochemical outcome models were additionally adjusted for baseline total energy intake (kcal/day), MVPA (min/day), and MedDiet score, all entered as continuous covariates. These covariates were selected a priori because dietary intake, physical activity, and overall diet quality may influence anthropometric and cardiometabolic biomarkers independently of the intervention.

Urinary phenolic metabolite models included intervention, period, sequence, and the corresponding baseline metabolite concentration as fixed effects, with participant as a random intercept. Additional lifestyle covariates were not included in these models because urinary phenolic metabolites were considered objective exposure biomarkers primarily reflecting the assigned oil intervention. This approach was also used to avoid unnecessary overadjustment and loss of precision given the modest sample size.

For outcomes analyzed on the logarithmic scale (HT-glucuronide, HT-sulfate, Tyr, Tyr-glucuronide), intervention effects were exponentiated and are presented as geometric mean ratios (GMRs) with 95% CI. For outcomes analyzed on the original scale (waist circumference, WHR, WHtR), results are presented as regression coefficients (β) with 95% CI.

As a supplementary analysis, FFQ-derived dietary intake was compared between intervention periods. For each dietary variable, post-intervention intake was analyzed using a linear mixed-effects ANCOVA model including intervention, the corresponding baseline intake, period, and sequence as fixed effects and participant as a random intercept. Adjusted EVOO–OO differences with 95% confidence intervals are reported together with period-specific descriptive values. The Benjamini–Hochberg procedure was applied separately to the 14 nutrient variables reported in Panel A and to the 10 selected food and beverage variables reported in Panel B Supplementary Table S4. Nonsignificant between-intervention comparisons were not interpreted as evidence of dietary equivalence.

No multiplicity-adjustment strategy was prespecified for the cardiometabolic or urinary phenolic metabolite outcomes reported in the present secondary analysis. Accordingly, *p*-values for these outcomes are reported as nominal and are interpreted in an exploratory framework, with emphasis on effect estimates, 95% confidence intervals, and consistency across sensitivity analyses. The Benjamini–Hochberg procedure was applied only to the supplementary FFQ-derived dietary analysis because this analysis involved simultaneous testing of multiple related dietary variables.

As an exploratory analysis, we examined whether within-period changes in urinary HT-glucuronide were associated with changes in the cardiometabolic outcomes that showed favorable between-intervention responses in the main analysis, namely HbA1c, waist circumference, WHR, and WHtR. Associations were assessed as continuous relationships using Spearman’s rank correlation coefficients, computed separately for the EVOO and OO periods.

Potential carryover was explored by comparing pre-intervention values at the beginning of the second period across randomization sequences using Wilcoxon rank-sum tests. Pre-period-2 HbA1c values did not differ significantly by sequence (*p* = 0.688); however, this comparison was not considered sufficient to exclude residual influence from the preceding intervention.

Post hoc sensitivity analyses were performed for waist circumference, HbA1c, and tyrosol-glucuronide. First-period-only analyses were conducted using data collected before participants crossed over to the alternate intervention. For waist circumference and HbA1c, between-group differences during the first intervention period were estimated using ANCOVA adjusted for the corresponding period-specific baseline value; the waist-circumference model was additionally adjusted for baseline MVPA, total energy intake, and Mediterranean diet adherence, consistent with the main cardiometabolic model. Tyrosol-glucuronide was analyzed on the logarithmic scale and adjusted for its corresponding baseline value, with the intervention effect back-transformed and expressed as a GMR. In addition, leave-one-participant-out influence analyses were performed by sequentially excluding each participant and refitting the corresponding full crossover model.

All tests were two-sided. Given the secondary and exploratory nature of the outcomes reported here, nominal *p*-values are presented alongside effect estimates and 95% confidence intervals and should not be interpreted as confirmatory evidence. All statistical analyses were performed using Stata software (version 14.0; StataCorp LP, College Station, TX, USA). Figures were generated using R statistical software (version 4.5.0; R Foundation for Statistical Computing, Vienna, Austria), with the dplyr, tidyr, ggplot2, ggtext, scales, and Hmisc packages.

## 3. Results

The intervention was well-tolerated by all participants, and no adverse events related to the study oils were reported during either treatment phase.

### 3.1. Baseline Characteristics

**Figure 1:**
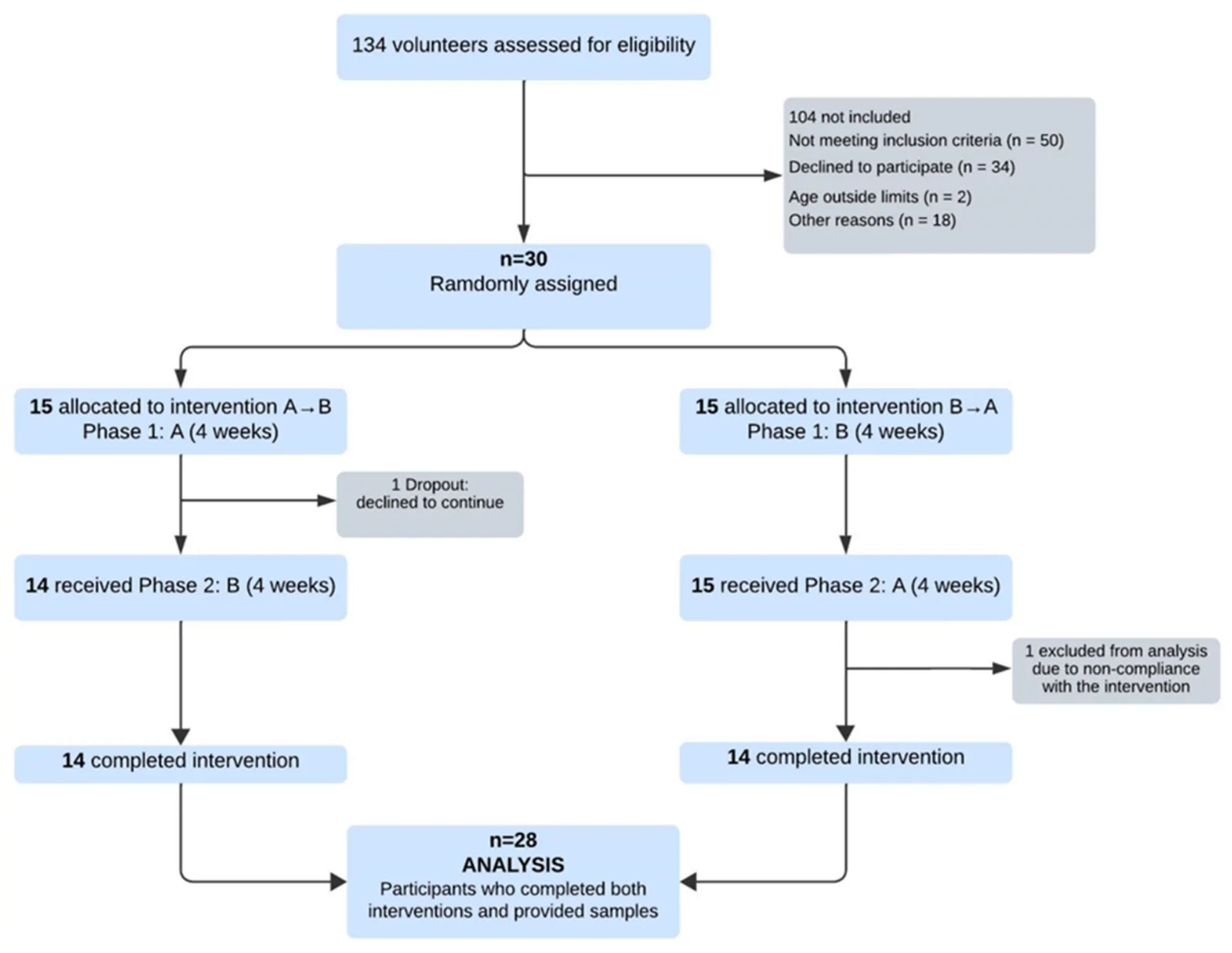
CONSORT flow diagram of participants through the HEVOOC randomized crossover trial.

Among the 134 volunteers assessed for eligibility, 30 met the inclusion criteria and were randomized to the intervention sequences. A total of 28 participants completed the study (Figure 1).

Baseline characteristics are presented in Table 1. Participants were young adults (29 ± 3.4 years), with normal anthropometric, biochemical, and blood pressure values; high physical activity levels; and an average energy intake of 2500 kcal/day.

Post-intervention dietary intake is presented in Table S4. Nominal between-intervention differences were observed for total protein, vegetable protein, fiber, and sugars; however, none of the 14 nutrient variables remained statistically significant after Benjamini–Hochberg correction. Adjusted between-intervention estimates and 95% confidence intervals are provided in Table S4 (Panel A). Available FFQ data on selected foods and beverages relevant to olive-oil phenolic exposure and the broader polyphenol-rich dietary background are also reported in the Supplementary Materials (Panel B). These comparisons are descriptive and should not be interpreted as evidence of formal dietary equivalence.

### 3.2. Body Composition

**Figure 2:**
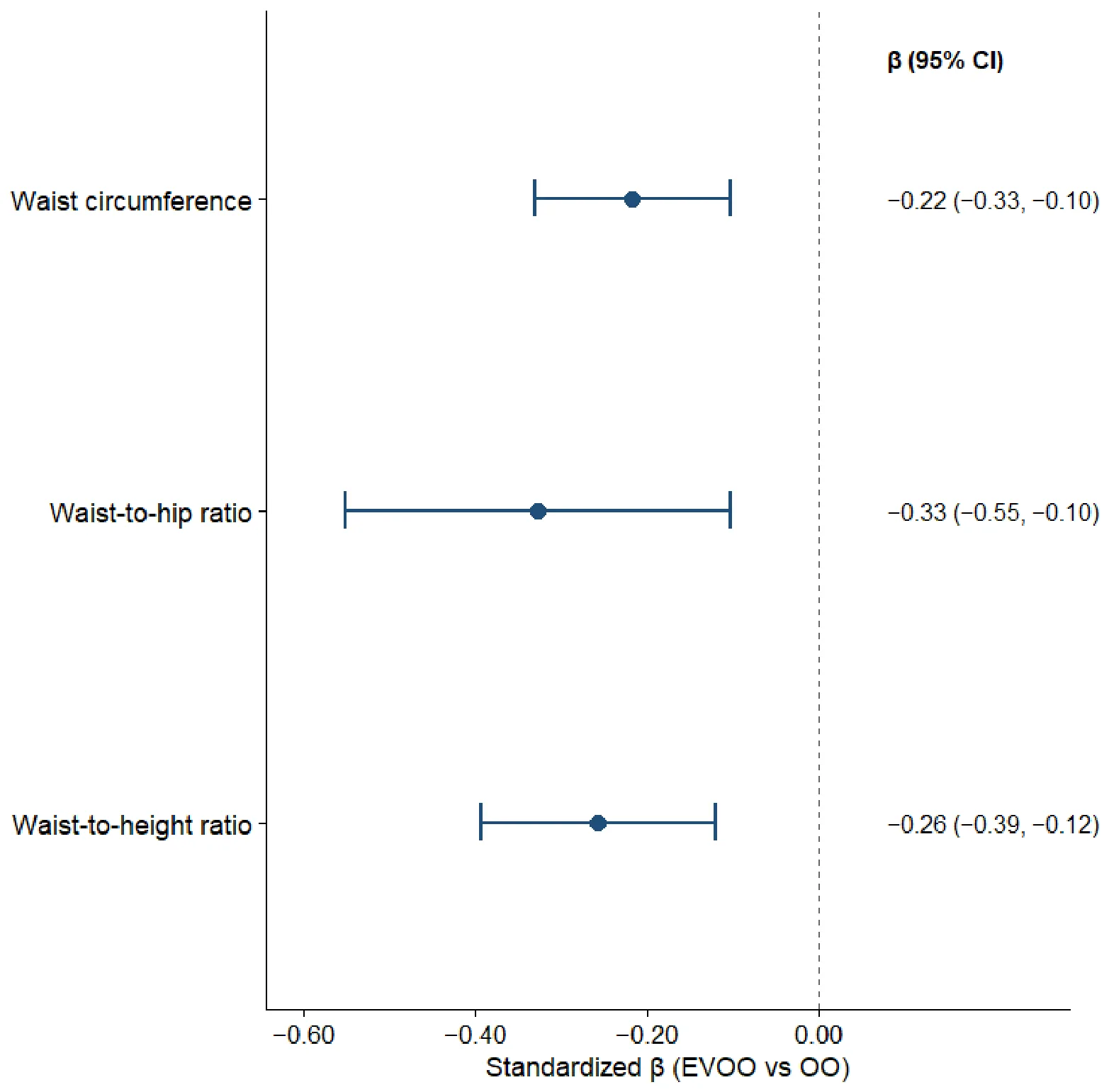
Treatment effects of EVOO vs. OO on central adiposity markers. Points represent standardized regression coefficients obtained from linear mixed-effects models fitted to z-transformed outcome variables, enabling comparison of relative effect magnitudes across outcomes measured on different scales. Unstandardized effect estimates are reported in the text. Models were adjusted for the corresponding baseline outcome value, period, sequence, baseline total energy intake, Mediterranean diet adherence, and physical activity.

Compared with OO, EVOO consumption was associated with lower central adiposity markers, including waist circumference (β = −2.21 cm; 95% CI: −3.37, −1.05; *p* < 0.001), WHR (β = −0.023; 95% CI: −0.039, −0.007; *p* = 0.004), and WHtR (β = −0.013; 95% CI: −0.020, −0.006; *p* < 0.001). Standardized effect estimates are presented in Figure 2. In contrast, body weight, BMI, total fat mass, muscle mass, and visceral fat did not differ significantly between treatments (Table S5).

Individual waist-circumference trajectories are shown in Figure S2. Descriptively, waist circumference decreased during the EVOO period in 19 of 28 participants and during the OO period in 11 of 28 participants; mean within-period changes were −1.54 cm and 0.64 cm, respectively. These descriptive data were not used as an independent test of the intervention effect.

### 3.3. Urinary Phenolic Metabolites

**Figure 3:**
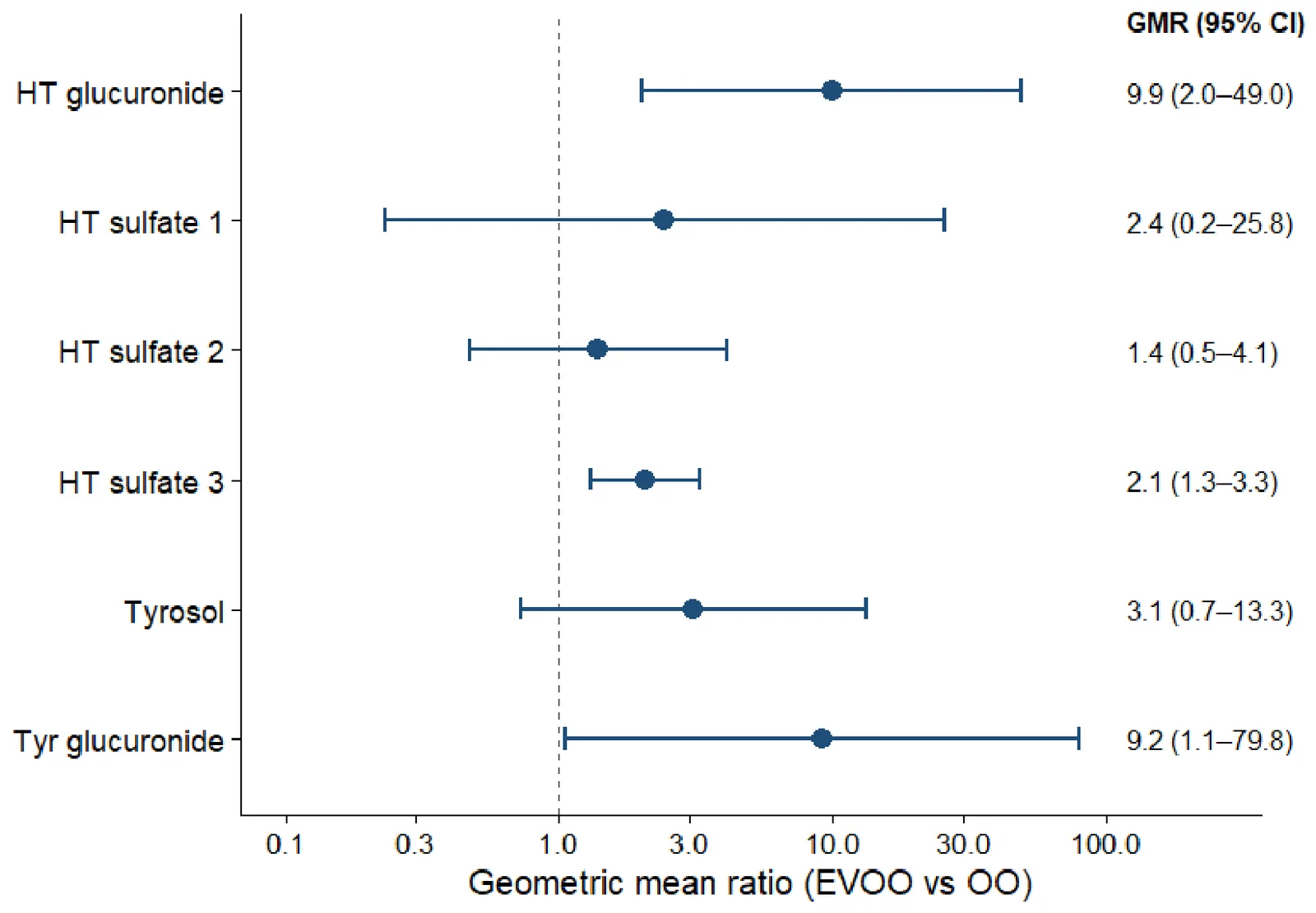
Treatment effects of EVOO compared with OO on urinary phenolic metabolites. The *x*-axis is displayed on a logarithmic scale, where the dashed vertical line at GMR = 1.0 indicates no treatment effect. Models were adjusted for period, sequence, and baseline metabolite concentrations. HT: Hydroxytyrosol; Tyr: Tyrosol.

Compared with OO, EVOO consumption resulted in higher urinary concentrations of HT glucuronide (GMR = 9.9; 95% CI: 2.0, 49.0; *p* = 0.005), HT-sulfate 3 (GMR = 2.1; 95% CI: 1.3, 3.3; *p* = 0.002), and tyrosol glucuronide (GMR = 9.2; 95% CI: 1.1, 79.8; *p* = 0.045). The corresponding GMRs and 95% CIs are shown in Figure 3.

### 3.4. Metabolic Biomarkers

EVOO was associated with lower HbA1c values compared with OO (β = −0.13%; 95% CI: −0.25, −0.01; *p* = 0.032). No significant differences between intervention were observed for fasting glucose, total cholesterol, triglycerides, LDL-C, HDL-C, ApoA1, or ApoB (Table S6). Changes in lipid parameters were modest and remained within clinically normal ranges throughout the study period.

### 3.5. Exploratory Analysis: Urinary Hydroxytyrosol-Glucuronide and Cardiometabolic Changes

**Figure 4:**
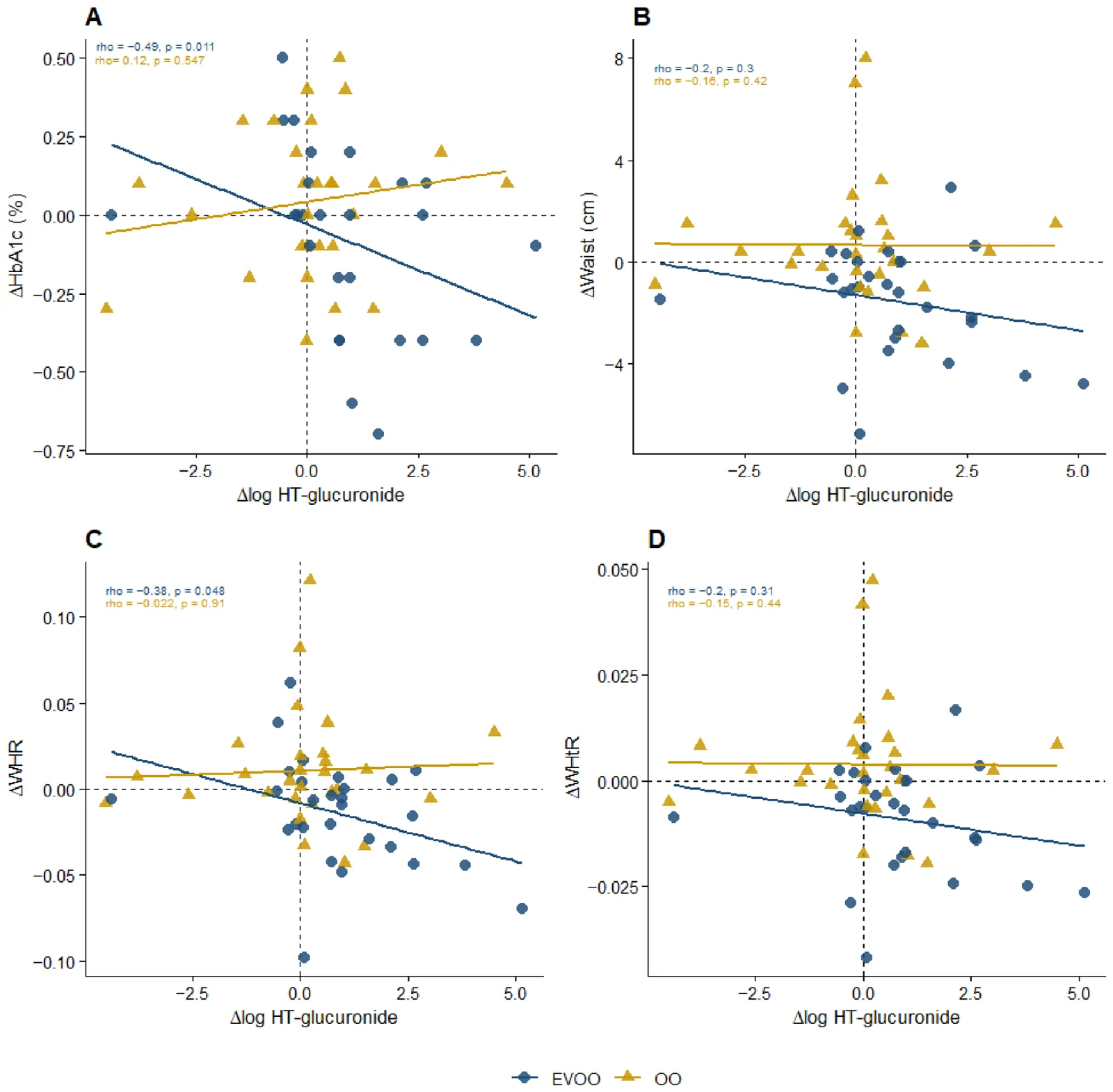
Exploratory correlations between changes in urinary hydroxytyrosol-glucuronide and changes in cardiometabolic outcomes. Scatterplots show the association between changes in urinary hydroxytyrosol-glucuronide (*x*-axis) and changes in (**A**) HbA1c, (**B**) waist circumference, (**C**) WHR (waist-to-hip ratio), and (**D**) WHtR (waist-to-height ratio) during the EVOO and OO periods. Correlation coefficients and *p*-values correspond to Spearman’s rank correlation analyses. Fitted lines are shown for visualization purposes only.

As an exploratory analysis, Spearman’s rank correlation coefficients were used to examine the associations between within-period changes in urinary hydroxytyrosol-glucuronide and changes in cardiometabolic outcomes (Figure 4). During the EVOO period, increases in urinary hydroxytyrosol-glucuronide were inversely correlated with decreases in HbA1c (Spearman’s rho = −0.49, *p* = 0.011) and with reductions in WHR (rho = −0.38, *p* = 0.048), whereas no significant associations were observed for waist circumference (rho = −0.20, *p* = 0.300) or WHtR (rho = −0.20, *p* = 0.310). In contrast, no significant correlations emerged during the OO period.

### 3.6. Post Hoc Sensitivity Analyses

Post hoc sensitivity analyses were performed for waist circumference, HbA1c, and tyrosol-glucuronide (Table S7). In the first-period-only analysis, the estimated between-group difference in waist circumference was −2.76 cm (95% CI: −5.13, −0.39; *p* = 0.025), similar in direction and magnitude to the full crossover estimate (β = −2.21 cm; 95% CI: −3.37, −1.05). Leave-one-participant-out estimates remained negative in all 28 iterations (range, −2.44, −1.91), with all 95% confidence intervals remaining below zero.

For HbA1c, the first-period-only estimate was smaller than that obtained in the full crossover analysis and was imprecise (β = −0.078 percentage points; 95% CI: −0.277, 0.121; *p* = 0.426; *n* = 26). Leave-one-participant-out estimates remained negative in all 28 iterations (range, −0.156, −0.097 percentage points), although the confidence interval included no between-treatment difference in some iterations. Two post-intervention HbA1c laboratory results during the EVOO period were unavailable despite collection of blood samples at the corresponding visits. No missing HbA1c values were imputed, and analyses were based on the available observations.

For tyrosol-glucuronide, the first-period-only analysis yielded a GMR = 3.83 (95% CI: 0.085, 173.25; *p* = 0.475). In the leave-one-participant-out analysis, all estimated GMRs remained above 1 (range, 6.19, 16.48), although confidence intervals included 1 in several iterations.

Overall, the waist-circumference estimate showed greater stability across sensitivity analyses, whereas the HbA1c and tyrosol-glucuronide estimates showed greater uncertainty.

## 4. Discussion

The present randomized crossover trial showed that short-term intake of phenolic-rich ‘Corbella’ EVOO, compared with low-polyphenol OO, produced a marked increase in urinary hydroxytyrosol- and tyrosol-derived metabolites and was associated with exploratory between-intervention differences in waist-related indices and HbA1c in healthy young adults. These findings support the use of urinary phenolic metabolites as objective exposure markers, while the cardiometabolic observations should be considered exploratory and hypothesis-generating.

Central adiposity is widely recognized as a sensitive early marker of cardiometabolic risk, often preceding overt alterations in body weight, lipid profile, or glycemic status [9]. In the present study reductions in waist circumference and waist-derived indices were observed despite the absence of significant between-intervention differences in body weight or total fat mass. This pattern may reflect differences in waist-based anthropometric measures in the absence of a detectable change in overall adiposity. Central adiposity is metabolically relevant and closely linked to cardiometabolic dysfunction [32], although detectable changes in visceral or total adiposity may require longer interventions or more sensitive assessment methods than those used in the present study. Given that central adiposity is a validated marker of cardiometabolic risk, waist-based measures may provide potentially informative cardiometabolic information even in metabolically healthy populations; however, the clinical relevance of the magnitude observed here remains uncertain [9]. Changes in waist circumference and body weight do not necessarily occur in parallel during dietary interventions. In the PREDIMED trial, an energy-unrestricted Mediterranean diet supplemented with EVOO was associated with no significant between-group difference in body weight and some evidence of less central adiposity compared with the control diet [8]. In the present study, however, the waist-circumference difference was not accompanied by corresponding differences in body weight, BMI, total body fat, or bioimpedance-derived visceral fat. Accordingly, this finding should not be interpreted as evidence of a reduction in abdominal or overall adiposity. Measurement variability, sampling variability, regression to the mean, and multiple testing cannot be excluded. Nevertheless, the direction and approximate magnitude of the waist-circumference estimate were maintained in the first-period-only and leave-one-participant-out sensitivity analyses, suggesting that the observed difference was not driven by a single participant. Given the short intervention period, modest sample size, and secondary nature of this outcome, confirmation in larger studies is required.

No significant between-intervention differences were observed for body weight or BMI. Thus, the waist-related findings were not accompanied by corresponding differences in overall body weight. This pattern should be interpreted cautiously given the short intervention duration and the absence of detectable differences in other measures of overall adiposity. This observation is consistent with large prospective cohort analyses showing that habitual olive oil consumption is not associated with long-term weight gain [33].

No significant between-intervention differences were detected in conventional lipid parameters after four weeks of intervention. The short intervention duration, normal baseline lipid concentrations, and modest sample size may have limited the ability to detect small between-intervention differences. Therefore, the present data do not provide evidence of a differential effect of EVOO versus OO on circulating lipid parameters under the conditions of this study.

As expected from the marked difference in phenolic content between the intervention oils, ‘Corbella’ EVOO produced substantially higher urinary concentrations of selected hydroxytyrosol- and tyrosol-derived metabolites. These findings are interpreted primarily as objective evidence of differential phenolic exposure between interventions rather than as a novel biological effect. The significant increases in hydroxytyrosol-glucuronide, hydroxytyrosol-sulfate, and tyrosol-glucuronide observed during the EVOO phase are consistent with the extensive phase II metabolism of hydroxytyrosol and tyrosol, mainly through glucuronidation and sulfation, which increases their water solubility and facilitates urinary excretion. Consequently, these conjugated metabolites represent the predominant circulating and urinary forms [17,18]. Hydroxytyrosol is rapidly absorbed and extensively metabolized following olive oil intake, reaching peak plasma concentrations within approximately 30 min and showing an estimated elimination half-life of approximately 2.4 h [34]. Its pharmacokinetics and bioavailability are influenced by the food matrix, with EVOO providing a favorable matrix for hydroxytyrosol bioavailability [18]. However, these observations do not establish prolonged systemic persistence of all EVOO-derived conjugated metabolites. In the present study, metabolite concentrations were measured in complete 24-h urine collections. Because serial plasma or fractionated urine samples were not obtained, pharmacokinetic parameters or the temporal profile of metabolite appearance and elimination could not be assessed.

These findings also support the use of hydroxytyrosol conjugates as objective adherence markers in EVOO interventions, complementing self-reported intake and strengthening exposure assessment in crossover designs where precise characterization of intervention exposure is important. The tyrosol-glucuronide estimate should nevertheless be interpreted cautiously. Although the direction of the estimated intervention effect remained above 1 in sensitivity analyses, both the main crossover and first-period-only estimates were imprecise, with particularly wide confidence intervals. This finding is therefore considered exploratory.

Potential mechanisms proposed in previous studies include modulation of postprandial glycemic responses, oxidative-stress-related pathways, and insulin signaling [10,11,35]. These mechanisms were not directly assessed in the present trial.

Participants had normal baseline HbA1c values (mean 5.1 ± 0.2%). Therefore, the small between-intervention difference observed in the main crossover analysis occurred within the normoglycemic range and should not be interpreted as evidence of a clinically meaningful improvement in glycemic control. Moreover, in the post hoc first-period-only analysis, the estimated effect was smaller, and the 95% confidence interval included no between-group difference. Leave-one-participant-out analyses maintained the direction of the estimate but showed some sensitivity in magnitude and precision. In addition, the HbA1c difference was not accompanied by corresponding between-intervention differences in fasting glucose, HOMA-IR, conventional lipid parameters, ApoA1, or ApoB, which limits the biological consistency of this finding. Given the longer time frame reflected by HbA1c, the short intervention and washout periods, the modest sample size, multiple secondary outcomes, and the exploratory nature of this finding, sampling variability and residual temporal influence cannot be excluded.

In the exploratory correlation analysis, greater increases in urinary HT-glucuronide during the EVOO period were associated with changes in HbA1c and WHR, whereas no significant associations were observed during the OO period or for waist circumference or WHtR. Because urinary hydroxytyrosol-derived metabolites can reflect exposure to EVOO phenolics [17,36], these findings suggest that participants with greater increases in this urinary exposure biomarker also tended to show more favorable changes in HbA1c and WHR. However, these associations do not establish that HT-glucuronide mediated these cardiometabolic changes. Previous evidence indicates that hydroxytyrosol and other olive oil phenolics may influence pathways related to oxidative stress, inflammation, and metabolic regulation [4,37,38]. In addition, EFSA concluded that olive-oil polyphenols contribute to the protection of blood lipids from oxidative stress under specified conditions of intake [39]. However, this external evidence does not demonstrate that these pathways were modified in the present trial, as oxidative stress, inflammatory pathways, and other potential mediating mechanisms were not directly assessed. Therefore, these correlations should be considered exploratory and hypothesis-generating rather than evidence of a causal or mechanistic relationship.

This study has several strengths. The randomized crossover design allowed each participant to serve as their own control, thereby reducing inter-individual variability and increasing the statistical efficiency of the analyses despite the modest sample size. Another strength is that the assigned oils were consumed as part of participants’ habitual meals, reflecting a real-world dietary exposure rather than an isolated supplement. In addition, the use of a body-weight-standardized oil dose improved internal validity. Outcomes were comprehensively assessed, including anthropometric, biochemical, dietary, physical activity, and urinary phenolic metabolite measurements. The use of urinary hydroxytyrosol-derived metabolites as objective biomarkers strengthens exposure assessment and reduces reliance on self-reported intake. Nevertheless, certain limitations should be acknowledged. The short intervention period and the relatively small sample size may have limited the ability to detect subtle changes in lipid and metabolic outcomes, particularly for secondary and exploratory endpoints. In addition, cardiometabolic outcomes were secondary endpoints; the trial sample size was originally calculated for another primary outcome. No multiplicity-adjustment strategy was prespecified for the heterogeneous cardiometabolic and urinary phenolic metabolite outcomes reported here; therefore, nominal *p*-values, particularly those close to 0.05, should be interpreted cautiously and as exploratory. Post hoc sensitivity analyses provided different degrees of support across outcomes: the waist-circumference estimate showed greater stability, whereas the HbA1c and tyrosol-glucuronide estimates showed substantially greater uncertainty. Waist circumference was based on a single measurement per study visit; although measurements were performed by the same trained evaluator, some within-observer measurement variability cannot be excluded.

Although longitudinal phenolic and oxidative-stability measurements were available for the ‘Corbella’ EVOO prototype under controlled storage conditions, these conditions did not fully reproduce individual household storage and culinary practices. The phenolic composition of residual oil from participants’ containers was not reassessed after each 4-week intervention period, and the phenolic content after individual cooking procedures was not measured. Therefore, some variability in the actual phenolic dose resulting from repeated opening, storage, and culinary use cannot be excluded. Comparable longitudinal stability measurements were not available for the control OO. Therefore, the observed effect estimates, particularly for waist circumference, may be imprecise, could overestimate the true population effect, and should be interpreted cautiously until replicated in larger, adequately powered trials. The inclusion of young, metabolically healthy adults may be considered both a strength and a limitation. Because most cardiometabolic parameters were already within healthy ranges, the potential for measurable improvement was limited. However, these findings should not be interpreted as evidence of reduced cardiometabolic disease risk, and whether larger or more clinically relevant effects would occur in higher-risk populations cannot be determined from the present study. Confirmation is therefore required in larger, adequately powered trials, including higher-risk populations.

## 5. Conclusions

In this randomized crossover trial, short-term intake of phenolic-rich ‘Corbella’ EVOO produced a marked increase in urinary hydroxytyrosol- and tyrosol-derived metabolites and was associated with exploratory between-intervention differences in waist-related indices and HbA1c in young and metabolically healthy adults. The increase in urinary phenolic metabolites provided objective evidence of substantially greater phenolic exposure during the EVOO intervention, complementing the self-reported adherence records.

The differences in waist-related indices were not accompanied by significant between-intervention differences in body weight, BMI, total fat mass, or visceral fat, while the HbA1c estimate showed greater uncertainty in post hoc sensitivity analyses. Given the short intervention period, modest sample size, lack of a prespecified multiplicity-adjustment strategy, secondary nature of the cardiometabolic endpoints, and low-risk profile of the participants, these observations should be interpreted as exploratory and hypothesis-generating rather than as definitive evidence of cardiometabolic benefit. Overall, the present study supports further investigation of phenolic-rich autochthonous EVOO cultivars in larger trials specifically powered for cardiometabolic outcomes.

## Figures and Tables

**Table 1:** Baseline characteristics of the study participants.

Characteristic	*n* = 28
**Age, (years); mean (SD)**	29.0 (3.4)
**Sex, n (%):**	
Male	14 (50%)
Female	14 (50%)
**Marital status; n (%)**	
Single	25 (89%)
Married	3 (11%)
**Educational level; n (%)**	
Higher education	8 (29%)
Postgraduate education	20 (71%)
**Country of birth; n (%)**	
Spain	13 (46%)
Other countries	15 (54%)
**Body composition**	
Weight, kg; mean (SD)	66.9 (12.6)
BMI, kg/m^2^; mean (SD)	23.2 (3.0)
Body fat, %; mean (SD)	25.0 (6.5)
Muscle mass, %; mean (SD)	34.5 (5.1)
Visceral fat; mean (SD)	5.6 (2.8)
Waist circumference (cm); mean (SD)	78.1 (9.8)
**Clinical parameters; mean (SD)**	
Systolic Blood Pressure (mmHg)	108.4 (11.3)
Diastolic Blood Pressure (mmHg)	70.9 (8.9)
Heart Rate (beats/min)	62.8 (9.1)
**Biochemical parameters; mean (SD)**	
Total cholesterol (mg/dL)	168.0 (33.1)
Triglycerides (mg/dL)	76.7 (46.9)
LDL-C (mg/dL)	95.9 (29.5)
HDL-C (mg/dL)	58.2 (18.6)
Glucose (mg/dL)	88.5 (6.7)
HbA1c (%)	5.1 (0.2)
**Lifestyle and dietary factors; mean (SD)**	
MVPA (min/day)	281.8 (55.2)
MedDiet score (17-point scale)	10.0 (3.4)
Total energy intake (kcal/day)	2478.5 (691.2)
Carbohydrates (g/day)	249.7 (97.7)
Protein (g/day)	98.8 (28.3)
Total fat (g/day)	116.2 (31.8)
Saturated fatty acids (g/day)	33.0 (11.1)
Monounsaturated fatty acids (g/day)	52.7 (14.6)
Polyunsaturated fatty acids (g/day)	21.7 (8.5)
Cholesterol (mg/day)	331.1 (208.5)

Values are expressed as mean ± standard deviation (SD) or n (%). Abbreviations: BMI, body mass index; TG, triglyceride; LDL-C, low-density lipoprotein cholesterol; HDL-C, high-density lipoprotein cholesterol; HbA1c, glycated hemoglobin; MVPA, moderate-to-vigorous physical activity; MedDiet, Mediterranean diet.

## Data Availability

The datasets generated during the current study are available from the corresponding author on reasonable request. Additional details regarding the study protocol and statistical analysis plan are also available from the corresponding author. Trial registration details and pre-specified outcomes can be accessed at ClinicalTrials.gov (NCT05898113).

## Supplementary Materials

The following supporting information can be downloaded at: https://www.mdpi.com/article/10.3390/antiox15081033/s1. Figure S1: Study design; Figure S2: Individual participant waist circumference values before and after each intervention period; Table S1: Phenolic compounds analyzed in EVOO; Table S2: Fatty acid composition (%) of the study oils; Table S3: Stability assessment of the ‘Corbella’ EVOO prototype developed for the HEVOOC intervention; Table S4: Dietary intake during the OO and EVOO intervention periods Table S5: Changes in body composition parameters after EVOO and OO interventions; Table S6: Changes in metabolic biomarkers after EVOO and OO interventions; Table S7: Post hoc sensitivity analyses for waist circumference, HbA1c, and urinary tyrosol-glucuronide.

## Abbreviations

The following abbreviations are used in this manuscript:

AUDIT	Alcohol Use Disorders Identification Test
BMI	Body Mass Index
EVOO	Extra Virgin Olive Oil
GMR	Geometric Mean Ratio
HbA1c	Glycated Hemoglobin
HDL-C	High-Density Lipoprotein Cholesterol
HEVOOC	Healthy Extra Virgin Olive Oil ‘Corbella’
HT	Hydroxytyrosol
IDIBAPS	Institut d’Investigacions Biomèdiques August Pi Sunyer
LC–MS	Liquid Chromatography–Mass Spectrometry
LMM	Linear Mixed-Effects Model
LDL-C	Low-Density Lipoprotein Cholesterol
MD	Mediterranean Diet
MEDAS	Mediterranean Diet Adherence Screener
MVPA	Moderate-to-Vigorous Physical Activity
OO	Olive Oil (low-polyphenol olive oil)
RCT	Randomized Controlled Trial
SD	Standard Deviation
TG	Triglycerides
Tyr	Tyrosol
WC	Waist Circumference
WHR	Waist-to-Hip Ratio
WHtR	Waist-to-Height Ratio
