# Supplementary material for: Corbella Extra Virgin Olive Oil, Urinary Phenolic Metabolites, and Exploratory Cardiometabolic Outcomes in Healthy Young Adults: The HEVOOC Trial
Source: Antioxidants (Basel). 2026 Aug 19;15(8):1033. doi: 10.3390/antiox15081033 (PMC13509589; doi:10.3390/antiox15081033)
Supplement: Supplementary file 1 [file antioxidants-15-01033-s001.zip › antioxidants-4461336-supplementary.pdf]

SUPPLEMENTARY MATERIALS

Supplementary figures:

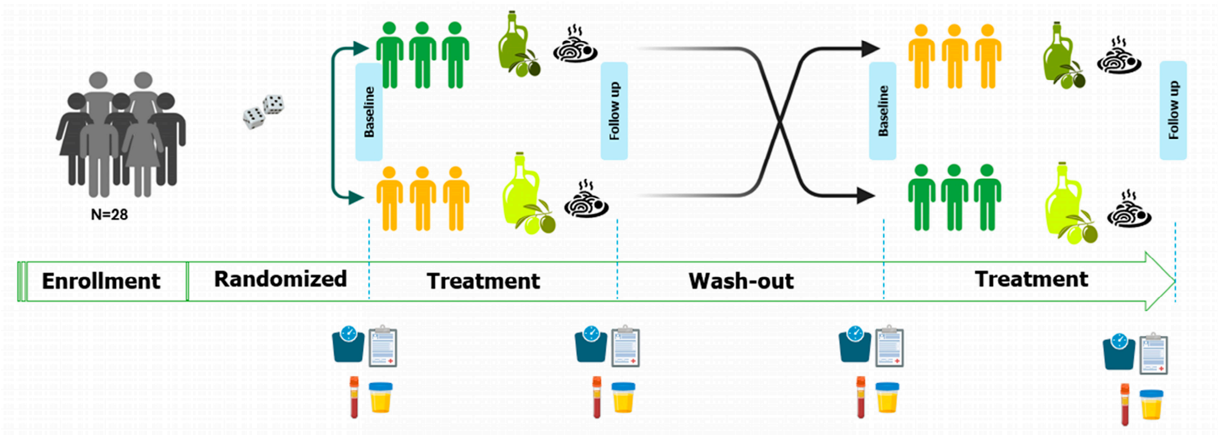

Figure S1. Study design. Overview of the clinical trial protocol, including participant allocation, intervention period, and sample collection timeline.

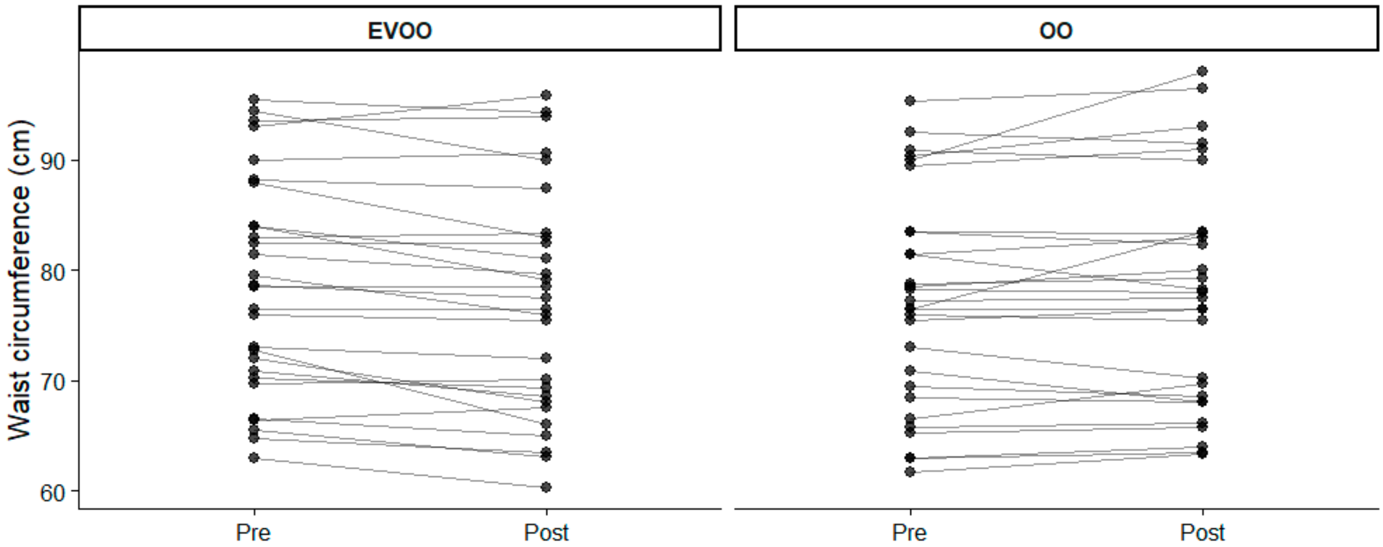

Figure S2. Individual participant waist circumference values before and after each intervention period. Each line represents one participant. The figure is descriptive; adjusted between-intervention estimates were obtained from the linear mixed-effects model described in the Methods.

Table S1. Phenolic compounds analyzed in EVOO

Phenolic compounds	Concentration (mg/kg)
Acetoxypinoresinol	0.605 ± 0.005
Apigenin	1.28 ± 0.03
DHOA	0.558 ± 0.005
Elenolic acid	3.5 ± 0.8
Ferulic acid	ULQ
HCM EA (isomer I)	0.581 ± 0.006
HCM EA (isomer II)	0.577 ± 0.005
HOA	0.974 ± 0.017
Hydroxyelenolic acid	0.621 ± 0.016
Isolariciresinol	3.7 ± 0.6
Lariciresinol	15.4 ± 1.4
Luteolin	ULQ
Methyl oleuropein aglycone	0.56 ± 0.005
Hydroxytyrosol	ULQ
Oleocanthalic acid	0.758 ± 0.019
Oleaceinic acid	0.569 ± 0.006
Oleuropein	0.558 ± 0.005
Pinoresinol	4.18 ± 0.09
Secoisolariciresinol	ULQ
Ligstroside aglycone	43.3 ± 1.7
Oleacein aglycone	46 ± 2
Oleocanthal	23.1 ± 1.3
Oleuropein aglycone	51 ± 3

Phenolic profile of the 'Corbella' EVOO used in the intervention. Values are expressed as mean ± SD (mg/kg), based on triplicate analyses. Phenolic compounds were quantified by UPLC–ESI–MS/MS using multiple reaction monitoring (MRM). ULQ, under the limit of quantification

Table S2. Fatty acid composition (%) of the study oils.

Fatty acid profile (%)	EVOO	OO
Myristic acid (C14:0)	0.00 ± 0.00	0.02
Pentadecanoic acid (C15:0)	0.02 ± 0.00	0.01
Palmitic acid (C16:0)	12.60 ± 0.70	11.8
Palmitoleic acid (C16:1 n7)	0.87 ± 0.00	1.18
Decenoic acid (C10:1 n9)	0.04 ± 0.00	n.r.
Margaric acid (C17:0)	0.03 ± 0.00	0.08
Heptadecenoic acid (C17:1 n7)	0.05 ± 0.00	n.r.
Stearic acid (C18:0)	2.14 ± 0.10	3.58
Oleic acid (C18:1 n9)	75.20 ± 1.60	74
Linoleic acid (C18:2 n6)	7.31 ± 0.01	7.1
Arachidic acid (C20:0)	0.30 ± 0.01	0.51
Linolenic acid (C18:3 n3)	0.56 ± 0.04	0.58
Gadoleic acid (C20:1 n9)	0.17 ± 0.00	0.28
Heneicosanoic acid (C21:0)	0.01 ± 0.00	0.04
Eicosadienoic acid (C20:2 n6)	0.02 ± 0.00	<0.01
Behenic acid (C22:0)	0.08 ± 0.00	0.16
Docosadienoic acid (C22:2 n6)	0.01 ± 0.00	n.r.
Lignoceric acid (C24:0)	0.05 ± 0.00	0.09

Values for the 'Corbella' EVOO are expressed as mean ± SD from replicate analyses. The fatty-acid profile of the 'Corbella' EVOO was determined by GC-FID after fatty-acid methyl ester derivatization. Values for the control OO were obtained from a single determination performed by an external laboratory and are therefore presented without SD.

Table S3. Stability assessment of the 'Corbella' EVOO prototype developed for the HEVOOC intervention.

Panel A. Oxidative-quality parameters

Parameter	Baseline	6 months Bag-in-box	6 months N_2	12 months Bag-in-box	12 months N_2
Rancimat, h at 110 °C	25.69 ± 0.23	24.69 ± 0.70	24.84 ± 0.29	24.75 ± 0.06	21.13 ± 0.28
1,2-DAGs, %	94.23 ± 0.06	83.27 ± 0.23	83.63 ± 0.15	68.77 ± 0.21	69.20 ± 0.26
PPPs, %	<0.5	2.13 ± 0.06	2.17 ± 0.21	6.77 ± 0.15	6.50 ± 0.20
Free acidity, % oleic acid	0.16 ± 0.02	0.20 ± 0.01	0.18 ± 0.01	0.19 ± 0.01	0.22 ± 0.01

Panel B. Major phenolic compounds

Compound, mg/kg	Baseline	6 months Bag-in-box	6 months N_2	12 months Bag-in-box	12 months N_2
Ligstroside aglycone	43.3 ± 1.7	57 ± 4	60 ± 3	45 ± 3	52 ± 2
Oleacein	46 ± 2	44 ± 3	48 ± 2	41.5 ± 1.7	53 ± 3
Oleocanthal	23.1 ± 1.3	24.3 ± 1.5	26.03 ± 1.12	17.9 ± 1.5	24.7 ± 1.4
Oleuropein aglycone	51 ± 3	59 ± 4	64 ± 3	56 ± 2	67 ± 4

Values are expressed as mean ± SD from triplicate analyses. The EVOO prototype was evaluated at baseline and after 6 and 12 months under bag-in-box and stainless-steel storage with N_2 in the headspace. Rancimat analyses were conducted at 110 °C. DAGs, 1,2-diacylglycerols; PPPs, pyropheophytins. Phenolic compounds were quantified by UPLC–ESI–MS/MS.

Table S4. Dietary intake during the OO and EVOO intervention periods

Panel A. Energy and nutrient intake

Nutrient	OO period, mean ± SD	EVOO period, mean ± SD	Adjusted difference (95% CI)	*p* value	*p* (BH)
Energy (kcal/day)	2453.7 ± 668.4	2476.8 ± 767.0	−118.5 (−250.4, 13.4)	0.076	0.175
Carbohydrates (g/day)	219.9 ± 86.6	224.3 ± 103.1	−16.9 (−36.3, 2.5)	0.085	0.175
Total protein (g/day)	93.4 ± 29.4	92.0 ± 30.6	−7.1 (−12.7, −1.5)	0.015	0.073
Animal protein (g/day)	55.1 ± 26.5	53.3 ± 27.0	−3.1 (−6.7, 0.4)	0.088	0.175
Vegetable protein (g/day)	38.3 ± 19.2	38.7 ± 23.1	−4.2 (−8.0, −0.5)	0.027	0.094
Total fat (g/day)	130.8 ± 31.6	131.9 ± 36.3	−1.5 (−8.9, 5.9)	0.680	0.794
Saturated fatty acids (g/day)	32.9 ± 10.6	33.4 ± 12.8	−0.6 (−3.1, 1.8)	0.611	0.794
Monounsaturated fatty acids (g/day)	67.2 ± 15.7	65.9 ± 18.4	−1.7 (−5.6, 2.0)	0.350	0.612
Polyunsaturated fatty acids (g/day)	21.5 ± 7.8	23.3 ± 8.1	0.1 (−1.8, 2.1)	0.878	0.913
Fiber (g/day)	33.9 ± 16.0	33.2 ± 17.5	−4.4 (−7.6, −1.1)	0.010	0.073
Polysaccharides (g/day)	122.7 ± 58.7	129.6 ± 78.1	−3.2 (−16.4, 9.9)	0.615	0.794
Sugars (g/day)	95.1 ± 38.3	92.6 ± 36.9	−8.5 (−15.2, −1.7)	0.016	0.073
Cholesterol (mg/day)	324.2 ± 184.8	322.7 ± 184.8	−1.3 (−25.7, 23.1)	0.913	0.913
Alcohol (g/day)	3.2 ± 3.9	3.5 ± 3.6	−0.3 (−1.7, 1.1)	0.642	0.794

Panel B. Selected food and beverage intake.

Selected dietary source	OO, mean ± SD	EVOO, mean ± SD	Adjusted difference (95% CI)	Nominal *p*	BH-adjusted *p*
Olive oil, g/day	47.9 ±10.8	48.6 ±11.6	0.87 (−2.90, 4.64)	0.638	0.808
Olives, times/week	0.00 ±0.00	0.05 ±0.15	0.05 (−0.006, 0.110)	0.079	0.396
Wine, g/day	11.0 ±24.2	9.4 ±18.2	−4.98 (−13.1, 3.16)	0.22	0.733
Red wine, g/day	5.78 ±13.6	4.7 ±8.8	−2.18 (−6.61, 2.26)	0.323	0.808
Beer, g/day	53.5 ±64.3	50.6 ±63.8	−4.90 (−20.9, 11.1)	0.534	0.808
Coffee, g/day	63.9 ±43.6	68.0 ±45.1	1.31 (−9.71, 12.3)	0.808	0.808
Tea, times/day	0.4 ±1.3	0.4 ±1.3	0.02 (−0.15, 0.20)	0.778	0.808
Chocolate, g/day	8.42 ±7.8	7.8 ±7.4	0.26 (−1.49, 2.01)	0.763	0.808
Fruit, g/day	209 ±151	172 ±117	−51.7 (−90.2, −13.2)	0.01	0.104
Vegetables, g/day	266 ±135	260 ±146	−10.7 (−45.9, 24.5)	0.537	0.808

Values for each intervention period are presented as mean ± SD. Adjusted differences represent EVOO minus OO estimates from linear mixed-effects models adjusted for the corresponding baseline dietary intake, period, and sequence, with participant included as a random intercept

Table S5. Changes in body composition parameters after EVOO and OO interventions

	EVOO intervention		OO intervention		Difference between interventions (EVOO vs OO) [a]	
**Body composition**	**Baseline (SD)**	**Mean change (95% CI)**	**Baseline (SD)**	**Mean change (95% CI)**	**Effect (95% CI)**	***p* value**
**Weight, kg**	67.2 (12.7)	0.3 (-0.0, 0.7)	67.2 (12.7)	0.5 (0.0, 0.8)	-0.17 (-0.66, 0.33)	0.508
**BMI, kg/m²**	23.4 (3.1)	0.1 (-0.1, 0.2)	23.4 (3.0)	0.2 (0.0, 0.3)	-0.07 (-0.25, 0.11)	0.439
**Body fat, %**	25.0 (6.3)	0.3 (-0.3, 0.9)	25.3 (6.7)	0.1 (-0.4, 0.6)	0.18 (-0.57, 0.93)	0.637
**Muscle mass, %**	34.6 (4.9)	-0.5 (-1.5, 0.4)	34.0 (5.2)	0.0 (-0.3, 0.3)	-0.45 (-1.33, 0.43)	0.316
**Visceral fat**	5.7 (2.8)	0.1 (-0.1, 0.3)	5.8 (2.9)	0.1 (-0.1, 0.3)	-0.00 (-0.27, 0.26)	0.990
**WC, cm**	78.6 (9.8)	-1.5 (-2.4, -0.7)	77.2 (9.8)	0.6 (-0.3, 1.6)	-2.21 (-3.37, -1.05)	**<0.001**
**WHR**	0.807 (0.065)	-0.014 (-0.026, -0.002)	0.790 (0.058)	0.011 (-0.002, 0.023)	-0.023 (-0.039, -0.007)	**0.004**
**WHtR**	0.465 (0.047)	-0.009 (-0.014, -0.004)	0.457 (0.049)	0.004 (-0.002, 0.009)	-0.013 (-0.020, -0.006)	**<0.001**

Data are presented as mean (SD) at baseline and mean change (95% CI) after each intervention. [a] Effect: estimated difference between interventions (EVOO vs. OO), derived from LMM adjusted for baseline values and covariates. Statistically significant difference between EVOO and OO interventions at $p < 0.05$. WC: waist circumference; WHR: waist-to-hip ratio; WHtR: waist-to-height ratio.

Table S6. Changes in metabolic biomarkers after EVOO and OO interventions

	EVOO Intervention		OO Intervention		Difference between interventions (EVOO vs OO) [a]	
	Baseline (SD)	Mean change (95% CI)	Baseline (SD)	Mean change (95% CI)	Effect (95% CI)	*p* value
TC (mg/dL)	169.6 (31.7)	2.8 (-3.7, 9.4)	165.4 (31.4)	4.2(-1.4, 9.9)	1.26 (-1.36, 3.88)	0.346
TG (mg/dL)	74.6 (39.6)	4.6 (-4.0, 13.3)	78.9 (48.5)	9.5 (-7.9, 26.9)	-3.6% (-10.7, 4.1) [b]	0.352
LDL-C (mg/dL)	95.6 (28.1)	0.8 (-3.9, 5.5)	92.3 (31.0)	2.1 (-3.4, 7.6)	0.51 (-1.71, 2.73)	0.654
HDL-C (mg/dL)	59.4 (18.6)	0.6 (-2.4, 3.6)	58.5 (15.9)	0.1 (-3.0, 3.3)	0.90 (-0.87, 2.68)	0.318
ApoA1	131.3 (25.3)	3.8 (-0.6, 8.3)	129.4 (19.6)	4.2(-0.6, 9.1)	0.73 (-2.02, 3.49)	0.602
ApoB	69.9 (18.0)	2.1 (-1.0, 5.4)	67.78 (19.2)	3.3 (1.1, 5.6) [a]	0.48 (-0.56, 1.51)	0.366
Glucose (mg/dL)	85.1 (6.6)	-2.4 (-5.8, 0.8)	86.3 (7.4)	-2.4 (-5.5, 0.6)	-0.98 (-3.45, 1.49)	0.438
HbA1c (%)[c]	5.0 (0.2)	-0.0 (-0.2, 0.0)	5.0 (0.2)	0.0 (-0.0, 0.1)	-0.13 (-0.25, -0.01)	**0.032***
HOMA-IR	1.5 (0.9)	-0.1(-0.3, 0.1)	1.4 (0.5)	-0.0 (-0.2, 0.1)	-6.8% (-22.3, 11.9) [b]	0.161

Data are presented as mean (SD) at baseline and mean change (95% CI) after each intervention.

[a] Effect: estimated difference between interventions (EVOO vs. OO), derived from LMM adjusted for baseline values and covariates. *Statistically significant difference between EVOO and OO interventions at $p < 0.05$

[b]Log-transformed variables were analyzed on the log scale and back-transformed for presentation; TG and HOMA-IR effects are expressed as percentage differences.

[c] HbA1c change during the EVOO period was available for 26 participants.

TC: total cholesterol; TG: triglycerides; LDL-C: low-density lipoprotein cholesterol; HDL-C: high-density lipoprotein cholesterol; ApoA1: apolipoprotein A1; ApoB: apolipoprotein B; HOMA-IR: homeostasis model assessment of insulin resistance.

Table S7. Post hoc sensitivity analyses for waist circumference, HbA1c, and urinary tyrosol-glucuronide

Outcome	Main crossover	First-period only	Leave-one-out range	Exclusion producing largest change	Maximum change
**Waist circumference (cm)**	−2.21 (−3.37, −1.05)	−2.76 (−5.13, −0.39)	−2.44 , −1.91	−1.91 (−3.01, −0.81)	**0.134**
**HbA1c (pp)**	−0.133 (−0.254, −0.011)	−0.078 (−0.277, 0.121)	−0.156 , −0.097	−0.097 (−0.212, 0.018)	**0.27**
**Tyr-glucuronide (GMR)**	9.17 (1.05, 79.84)	3.83 (0.08, 173.25)	6.19 , 16.48	16.48 (2.60, 104.50)	**26.5% ***

Values for full crossover and first-period-only analyses are effect estimates (95% CI). The leave-one-participant-out range represents the minimum and maximum treatment estimates obtained after sequential exclusion of each participant. *For tyrosol-glucuronide, the maximum relative change was calculated on the log-scale treatment coefficient rather than directly from the GMR. GMR, geometric mean ratio.
